# Topical Toxicity and Repellency Profiles of 17 Essential Oil Components against Insecticide-Resistant and Susceptible Strains of Adult *Musca domestica* (Diptera: Muscidae)

**DOI:** 10.3390/insects15060384

**Published:** 2024-05-24

**Authors:** Yuexun Tian, Jerome A. Hogsette, Edmund J. Norris, Xing Ping Hu

**Affiliations:** 1 Department of Entomology and Plant Pathology, Auburn University, Auburn, AL 36849, USA; yuexun.tian@ag.tamu.edu; 2 USDA-ARS, Center for Medical, Agricultural and Veterinary Entomology, Gainesville, FL 32608, USA; edmund.norris@usda.gov

**Keywords:** house fly, Y-tube-olfactometer, lethal dose, dose-response, attractant

## Abstract

**Simple Summary:**

The house fly is a pest that not only causes economic losses but also threatens human health, posing nuisance problems and mechanically transmitting pathogens that cause diseases. Although the use of pesticides has provided efficient control, resistance development has been hindering the success of traditional pesticides. In this study, we evaluated 17 essential oil components (EOCs) for their toxicity and repellency against the strains of resistant and susceptible house flies. Thymol, (+)-pulegone, eugenol, and carvacrol exhibited high toxicity to house flies with little to no resistance to the tested strains. Seven of the EOCs exhibited repellent activity towards house flies at certain concentrations. Two EOCs were attractive to house flies and can potentially serve as attractants for traps. Thymol could potentially be used as a lure and insecticide as it exhibits high toxicity and is attractive to house flies. Our screening of a wide range of EOCs provides a deeper understanding of the potential of using EOCs not only as pesticides but also as trap attractants, encouraging further investigations in house fly management using those EOCs.

**Abstract:**

The house fly is a significant pest in agriculture and human health that is increasingly difficult to manage due to multiple limitations including resistance development. To explore alternative pesticides, the topical toxicity and repellency profiles of 17 essential oil components (EOCs) were evaluated against a resistant and a susceptible strain of house fly, *Musca domestica* L., using topical application and Y-tube olfactometers, respectively. Six of the most toxic EOCs based on the LD_50_ were further investigated against a susceptible strain of house fly. Thymol, (+)-pulegone, eugenol, and carvacrol were always the top four most toxic chemicals tested against the resistant house fly strain. Little to no resistance was observed to the top six EOCs based on the comparison of the results between resistant and susceptible house fly strains. P-Cymene, citronellic acid, R-(+)-limonene, linalool, γ-terpinene, estragole, and eugenol were repellent to adult house flies at certain concentrations while (-)-carvone and thymol were attractive to adult house flies. This screening of a wide variety of individual EOCs provides a stronger foundation of information for further research. This should encourage further investigation into the topical toxicity and repellency in field studies, which will provide more insight into the performance of biopesticides for house fly management and potential commercialization.

## 1. Introduction

The house fly, *Musca domestica* L., is a widespread urban and public health pest of global importance [1]. House flies are associated with synanthropic ecosystems and propagate throughout the year with a high reproductive rate [2]. Adult flies pose nuisance problems to farm workers and neighboring residents. More importantly, they are medical and veterinary pests. Contaminated flies disperse to areas of human and other animal habitation and activity and mechanically transmit pathogens through their contacting, defecating, and regurgitating behaviors. A conservative estimate is that house flies are associated with the transmission of more than 100 etiological agents of bacterial, protozoan, and viral diseases [3,4], including metazoan parasites [5]. A detailed summary of the human pathogens carried by the house fly is summarized by Khamesipour et al. [6]. Additionally, they are intermediate hosts of parasitic nematodes of horses and some cestodes of poultry [7], such as *Draschia megastoma*, *Habronema majus*, *H. muscae* [8], and *Choanotaenia infundibulum* [9]. 

House fly management increasingly relies on integrated pest management (IPM), which involves a combination of various cultural methods, mechanical and trapping techniques, chemicals, and biological agents. However, IPM is sometimes difficult to implement because of the high labor costs and limitations of trapping methods and biological agents. Historically, chemical insecticides provided fast and full control with two primary strategies: reducing the fly population and reducing contact of flies with humans and other animals. Therefore, the toxicity, e.g., contact and fumigant toxicity, and repellency of the chemical insecticides greatly affect the efficiency of house fly control. However, if used improperly, insecticides can poison animals including humans, contaminate food and water, limit the efficacy of biological control agents [10,11], and increase the resistance levels of exposed fly populations. 

The house fly has shown a particular ability to develop resistance to both conventional and novel insecticides [12]. With a global increase in resistant house fly populations, interest in biopesticides has extended into botanical essential oils (EOs) and essential oil components (EOCs) as low-risk alternatives to synthetic insecticides [13,14,15]. It has been demonstrated that EOCs have multiple modes of action and affect a wide range of economically important insects, as well as tick and mite pests [16,17]. EOCs are generally known to have contact and fumigant insecticidal and repellent properties [18] and have traditionally been used to protect stored grain products from pests [19] and repel mosquitoes in residences [20]. From 1980 to 2011, the proportion of insecticidal studies focused on botanical insecticides rose from 2% to more than 21% [21]. 

Studies have reported the effects of some EOs and EOCs on house flies with different modes of action. For example, thyme oil and its major component thymol exhibited contact and toxicity to house fly larvae and prevented adults from emerging from pupae [22]. Thymol was demonstrated to be effective against adult house flies with a synergetic effect when mixed with p-cymene, another main component of thyme oil [23]. Evaluations were also conducted on essential oils of star anise and lemongrass for their contact toxicity and repellency with no harm to non-target organisms [24,25]. There also are studies that reported insecticidal effects on other dipterans, such as *Ceratitis capitata* [26] and *Drosophila suzukii* [15]. However, the level of toxicity and fly-control efficacy vary greatly among reports. Furthermore, some EOCs, which have been reported as highly toxic to various pest insects, have not been evaluated against house flies. 

The purpose of this study was to comparatively evaluate the topical toxicity and repellency of 17 selected individual EOCs against adult house flies using topical application and an olfactometer. Using these methods will allow comparisons to be made between this and other studies. A subset of these 17 EOCs has been evaluated previously on house flies, but under different conditions (Supplementary Table S1). We also investigated the relationship between their toxicological and chemical properties. Results of this study should provide insight into discovery of active ingredients and improvement of formulations to increase the performance of biopesticides for house fly management. 

## 2. Materials and Methods

### 2.1. Chemicals

The 17 individual EOCs, obtained from Sigma-Aldrich (St. Louis, MO, USA), were p-cymene (97%), γ-terpinene (99%), thymol (99%), eugenol (98%), geraniol (98%), linalool (97%), (1S)-(-)-verbenone (93%), methyl salicylate (99%), citronellic acid (98%), benzaldehyde (99.5%), (-)-carvone (98%), (+)-fenchone (98%), estragole (99%), (+)-pulegone (99%), carvacrol (98%), camphor (96%), and (R)-(+)-limonene (97%) (Supplementary Table S2).

### 2.2. House Flies

Permethrin-resistant and -susceptible laboratory-reared Florida house fly strains used for the bioassays were obtained from the U.S. Department of Agriculture, CMAVE, Gainesville, FL. The resistant strain was composed of flies collected in and around Gainesville, FL, where permethrin resistance has been reported to be high [27]. These flies were considered representative of local multi-resistant house fly populations. The susceptible strain was colonized by the USDA in the early 1950s to 1960s and has since been the standard for many studies. These flies have never been exposed to any pesticides [27]. Pupae from these two strains were shipped to Auburn University overnight and immediately placed in two separate uncovered plastic Petri dishes (2.5 cm × 15 cm dia., Becton Dickinson, Franklin Lakes, NJ, USA). Each Petri dish was placed inside a screen cage (30 cm^3^) and adults emerged within 2–3 days. Unenclosed pupae were removed from each cage at the end of the third day. Adult flies maintained in the cages were provided ad libitum with water and a diet of powdered milk, sugar, and dehydrated whole egg (2:2:1) [28]. The pupae and the adult house flies were maintained under laboratory conditions (25 ± 2 °C, 50–70% RH).

### 2.3. Topical Application

#### 2.3.1. Resistant House Fly Strain

Acute topical toxicity was evaluated using a modified method described by Pavela [29]. Caged house flies (3–5 days after eclosion) were anesthetized by placing the cage in a refrigerator (7–8 °C) for 15 min. A blank sheet of paper was placed at the bottom of the cage before it was placed in the refrigerator. As flies became anesthetized and fell onto the paper, they were quickly transferred into a metal pan (30 × 16 × 5 cm^3^) surrounded by ice to prevent their recovery. Female flies, identified by the relatively wide space between their compound eyes [30], were selected and immediately placed in glass Petri dishes (10.0 cm dia. × 1.5 cm high, Thermo Fisher Scientific, Waltham, MA, USA) in groups of 25.

Concentrations of EOCs that would cause 10–90% mortality, based on the results of preliminary concentration tests, were selected. Dilutions (5–7) of each test chemical were prepared in acetone (Avantor Performance Materials, Inc., Radnor Township, PA, USA). One microliter of each concentration was applied to the dorsal pronotum of each re-anesthetized female fly using a micro-applicator with a 25 μL gastight syringe (Hamilton Co., Reno, NV, USA). Acetone was used as the control treatment. Treated flies in groups of 25 were transferred to glass jars (9 cm dia. × 18 cm high) immediately after application with mesh placed on the top to prevent escape and facilitate airflow. A cotton ball soaked in a 10% sugar solution was placed at the bottom of the jar as the food source. Jars containing the treated flies were maintained under laboratory conditions (25 ± 2 °C, 50–70% RH), and mortality was recorded at 24 and 48 h posttreatment. A fly was defined as dead when it no longer exhibited movement after being prodded using a small brush. Each treatment was replicated four times. Replications with a control mortality rate exceeding 10% were discarded and repeated.

#### 2.3.2. Susceptible House Fly Strain

EOCs ranked in the top five in the resistant house fly strain mortality evaluations (lethal dose to 50 and 95% mortality at 24 and 48 h posttreatment) were evaluated to determine their acute topical toxicity levels on the susceptible house fly strain using the method described above. Dilutions (5–6) of each EOC were prepared. Twenty-five susceptible female house flies were tested for each dilution and maintained as described above.

### 2.4. Repellency Bioassay

Repellency bioassays were performed using a Y-tube olfactometer modified from Haselton et al. [31] and Liu et al. [32]. The Y-tube olfactometer consisted of a central arm and two lateral arms (each 20 cm long × 25 mm dia.), with removable glass adaptors located at the ends of all three arms. Two air streams, one for each lateral arm, were produced by an electric pump (Hydrofarm, Inc., Petaluma, CA, USA) and monitored by two flow meters. Before the air entered the lateral arms, each air stream was purified and humidified by passing it through a charcoal filter and bubbling it through tap water in a 100 mL flask. When the bioassays were conducted, pressurized air was constantly introduced into the lateral arms of the olfactometer through the odor source flasks at a rate of 220 mL/min. The airflow rate in the lateral arms was maintained at 200 mL/min. A 2 cm-diameter screen disk was placed inside and near the end of each lateral arm to prevent flies from entering the tubing in the olfactometer. To minimize visual distraction to the flies, the Y-tube olfactometer was oriented vertically in a room with overhead fluorescent lights on. The olfactometer was placed inside a cardboard box (82 × 82 × 61 cm high) with three of the four vertical walls lined with white paper. The top and the front sides of the box were opened for illumination and observation, respectively. 

EOCs were serially diluted in acetone to obtain five concentrations (100, 10, 1, 0.1, and 0.01 μg/μL). A 10 μL volume of each concentration was pipetted onto individual 1 × 2 cm filter paper strips (Fisher Scientific, Pittsburgh, PA, USA) and the solvent was allowed to evaporate for 30 s. A treated filter paper strip was inserted into one odor-source flask, and a control filter paper strip treated with the same volume of acetone was placed in the other odor-source flask. Odor source flasks with filter paper were placed on the ends of the lateral arms of the olfactometer. After waiting 30 s to allow the volatile chemicals of the EOCs to reach the central arm, a single house fly was transferred to the central arm. Each fly was observed for 2 min. If it moved into one of the lateral arms, crossing a line drawn on the arm 10 cm above the intersection of the arms and remained there for at least 15 s, its choice was recorded. Flies remaining in the central tube or not crossing the lines on the lateral arms were not counted. Fifteen flies of each sex were tested for each treatment (concentration and chemical) and flies were only used once. The olfactometer was rotated 180° along the long axis of the central arm after every five flies to minimize any positional bias in the lateral arms. After 10 flies (5 females and 5 males) were tested, which required 7–10 min, the treated and control filter paper strips were discarded and replaced with new strips treated with the same chemical and concentration. After tests were completed, the olfactometer apparatus was washed thoroughly with soapy water, rinsed with 95% ethanol, and air-dried. All the bioassays were conducted under laboratory conditions (25 ± 2 °C and 50–70% RH).

### 2.5. Data Analysis

Probit analysis was used to estimate the 50% and 95% mortality values (LD_50_ and LD_95_) and the 95% confidence limits (PoloPlus, 2003; LeOra Software LLC, Parma, OH, USA. https://leora-software.com/, accessed on 15 May 2024). Non-overlapping 95% confidence limits were used to determine significant differences among LD_50_ and LD_95_ values. Vapor pressure data were obtained from the Environmental Protection Agency Toxicity Estimation Software Tool (T.E.S.T.) similar to Norris et al. [33]. Empirical data were used for each EOC when available (as provided and denoted by T.E.S.T software v. 5.1.2); otherwise, predicted vapor pressure values (provided by the software) were used. As an exponential relationship between raw toxicity and vapor pressure was apparent, vapor pressure and toxicity (LD_50_ values) data were log-transformed and plotted. Pearson correlation analysis was then used to assess the degree of correlation between both variables. For the repellency tests, the preference of fly choice was calculated using the following equation:

$$Preference(\%)=\frac{Number\;of\;fly\;chosing\;the\;arm\;(control/treatment)}{Total\;numbers\;of\;lies\;tested}$$

Chi-square tests (SPSS 17.0, SPSS Inc., Chicago, IL, USA) were used to test the null hypothesis that there was no preference for house flies between the treatment and control. 

## 3. Results

### 3.1. Topical Application

At 24 h posttreatment, the LD_50_ values of the 17 individual EOCs against females of the resistant house fly strain ranged from 43.8 to 512.1 µg/fly (Table 1). Thymol was the most toxic component with an LD_50_ of 43.8 µg/fly followed by (+)-pulegone (73.0 µg/fly), eugenol (89.5 µg/fly), carvacrol (90.8 µg/fly), and citronellic acid (93.4 µg/fly). Camphor was the least toxic with an LD_50_ of 512.1 µg/fly followed by (1S)-(-)-verbenone (426.7 µg/fly) and (+)-fenchone (405.1 µg/fly). Thymol was significantly more toxic, and camphor was significantly less toxic than all other EOCs tested. 

At 24 h posttreatment, the LD_95_ values of the 17 individual EOCs against females of the resistant house fly strain ranged from 155.6 to 1322.1 µg/fly (Table 1). (+)-Pulegone was the most toxic compound with an LD_95_ of 155.6 µg/fly, followed by eugenol (182.9 µg/fly), carvacrol (275.7 µg/fly), thymol (360.4 µg/fly) and benzaldehyde (392.4 µg/fly). Linalool was the least toxic component with an LD_95_ of 1322.1 µg/fly followed by (R)-(+)-limonene (1208.5 µg/fly) and (+)-fenchone (1094.9 µg/fly). 

At 48 h posttreatment, the LD_50_ values of the 17 individual EOCs against females of the resistant house fly strain ranged from 41.1 to 477.9 µg/fly (Table 2). Thymol was the most toxic with an LD_50_ of 41.1µg/fly, which was significantly lower than all others. Thymol was followed by (+)-pulegone (68.2 µg/fly), eugenol (78.5 µg/fly), carvacrol (80.6 µg/fly), and geraniol (85.8 µg/fly). Camphor was the least toxic with an LD_50_ of 477.9 µg/fly, followed by (1S)-(-)-verbenone (409.9 µg/fly) and (+)-fenchone (385.3 µg/fly). All LD_50_ values at 48 h posttreatment were slightly lower than the LD_50_ values at 24 h posttreatment. 

At 48 h posttreatment, the LD_95_ values ranged from 104.8 to 1356.9 µg/fly against females of the resistant house fly strain (Table 2). (+)-Pulegone was the most toxic component with an LD_95_ of 104.8 µg/fly, followed by eugenol (153.1 µg/fly), carvacrol (237.7 µg/fly), thymol (317.2 µg/fly) and geraniol (373.0 µg/fly). (R)-(+)-Limonene was the least toxic component with an LD_95_ of 1356.9 µg/fly, followed by (+)-fenchone (1138.6 µg/fly) and linalool (1042.4 µg/fly). (+)-Pulegone was significantly more effective than the other components tested. All LD_95_ values at 48 h posttreatment were slightly lower than the LD_95_ values at 24 h posttreatment except for estragole, (+)-fenchone, and limonene. The LD_95_ values for citronellic acid at 24 and 48 h posttreatment were essentially the same (Table 1 and Table 2).

The slope of the regression line indicates how fast the mortality increases as the dosage increases. For the resistant flies at 24 h posttreatment, thymol had the lowest LD_50_ and the lowest slope of 1.8, while camphor had the highest slope of 9.2 and the highest LD_50_ (Table 1). This indicates that to achieve higher mortality, thymol requires a greater increase in chemical dosage than camphor. Similar results were also observed at 48 h posttreatment (Table 2).

**Figure 1:**
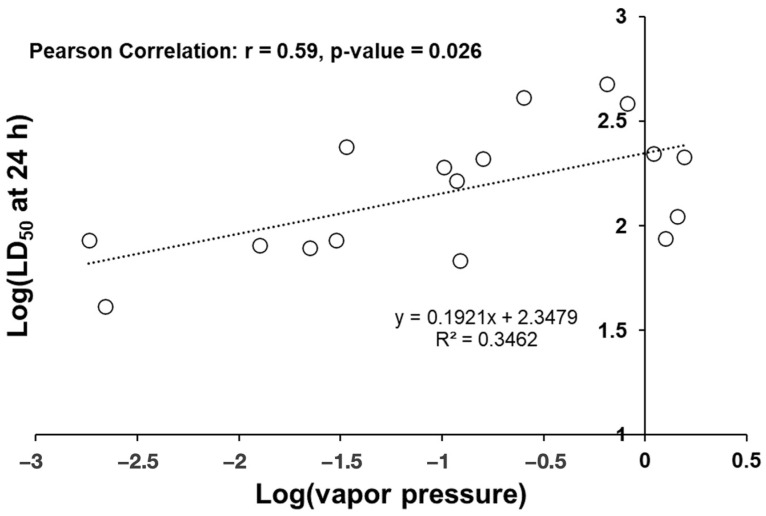
The 24 h LD_50_ values (log_10_-adjusted) for each essential oil component vs. vapor pressure.

A moderate correlation was observed (Pearson correlation r = 0.59, *p*-value = 0.026) between the toxicity of EOCs and their vapor pressures (mmHg) (Figure 1). Data were log-adjusted because of an apparent exponential relationship when plotting the raw data. Correlation indicates that the toxicity of the essential oil component is exponentially related to its vapor pressure, suggesting that low-toxicity essential oil constituents may volatilize before diffusing through the insect cuticle.

The top five most toxic EOCs from the resistant house fly strain based on LD_50_ values at 24 and 48 h posttreatment overlapped and resulted in the selection of six EOCs: thymol, (+)-pulegone, eugenol, carvacrol, citronellic acid and geraniol. When these were tested against females of the susceptible house fly strain, the LD_50_ values at 24 h posttreatment ranged from 35.2 to 93.1 µg/fly (Table 3). Thymol had the lowest LD_50_ value, but it was not significant. There were no significant differences at 24 h posttreatment between susceptible (Table 3) and resistant house flies (Table 1). At 48 h posttreatment, the LD_50_ values for females of the susceptible strain ranged from 29.2 to 75.7 µg/fly (Table 4). Thymol had the lowest LD_50_, which was significantly lower than that of citronellic acid. The LD_50_ values of (+)-pulegone and eugenol in the susceptible house fly tests (Table 4) were significantly lower than the LD_50_ values in the resistant house fly tests (Table 2).

At 24 h posttreatment, the LD_95_ values of the above six chemicals against females of the susceptible house fly strain ranged from 227.5 to 530.1 µg/fly (Table 3). Thymol was the most active component, and there was no significant difference between susceptible (Table 3) and resistant (Table 1) house flies. At 48 h posttreatment, the LD_95_ values for the susceptible females ranged from 207.2 to 479.4 µg/fly (Table 4). The LD_95_ value of (+)-pulegone was significantly higher in the susceptible house flies than the LD_95_ value in the resistant house flies, but there were no significant differences among the remaining chemicals.

### 3.2. Repellency Bioassay

**Figure 2:**
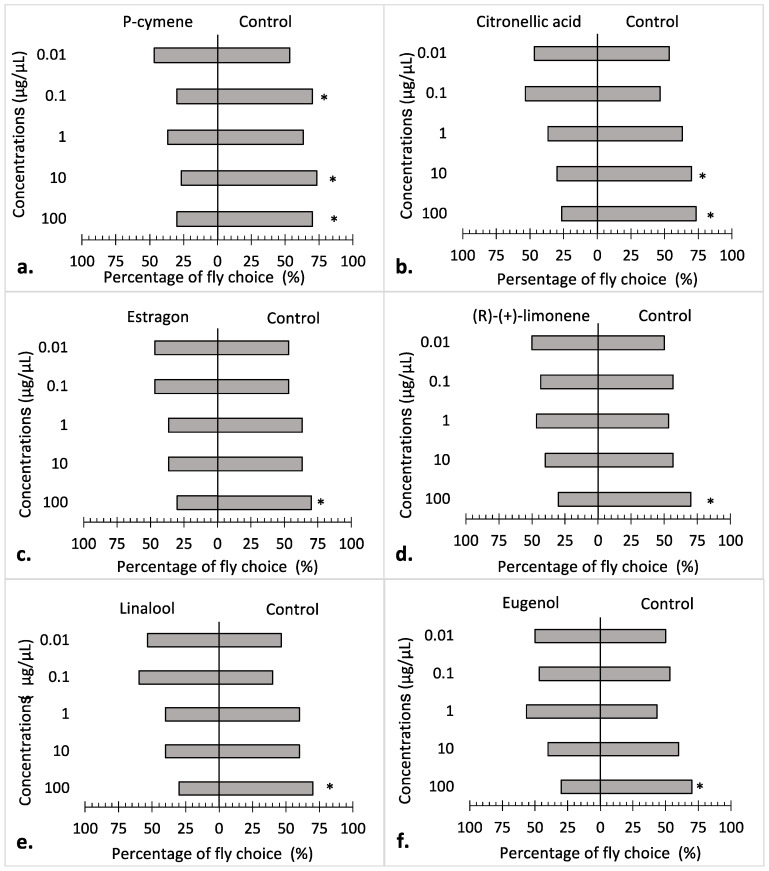

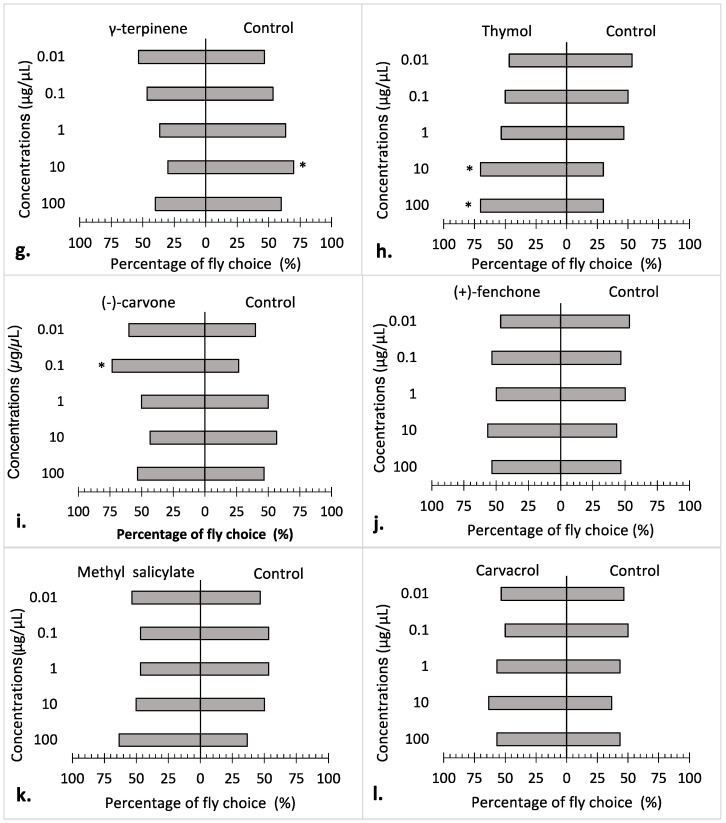

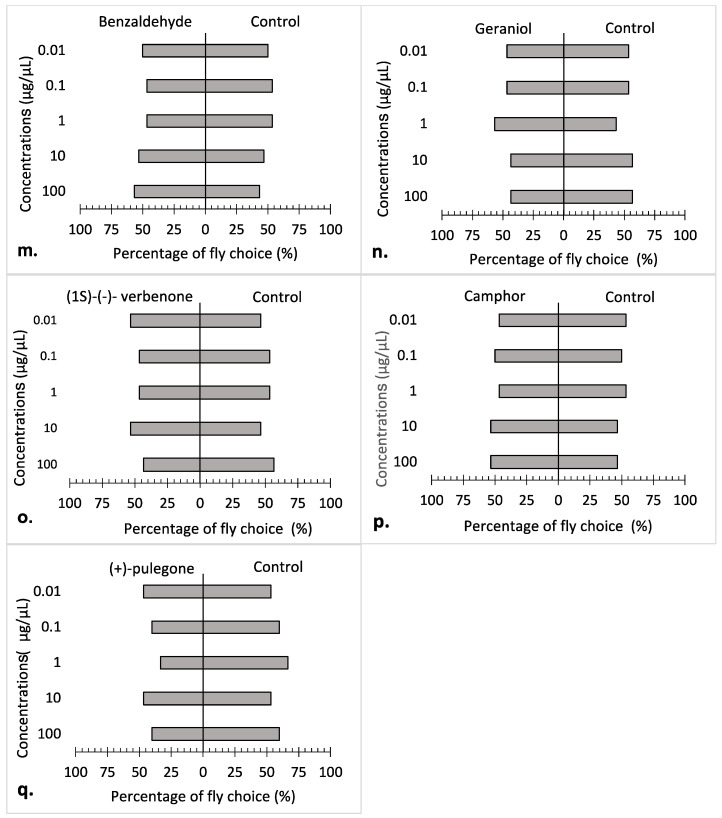
Behavioral responses of adult (*n* = 30) *Musca domestica* to 10 µL of essential oil components at different concentrations (0.01–100.00 µg/µL) in the Y-tube olfactometer bioassays. Asterisks (*) indicate a significant difference at *p* < 0.05. (**a**) P-cymene; (**b**) Citronellic acid; (**c**) Estragon; (**d**) (R)-(+)-limonene; (**e**) Linalool; (**f**) Eugenol; (**g**) γ-terpinene; (**h**) Thymol; (**i**) (-)-carvone; (**j**) (+)-fenchone; (**k**) Methyl salicylate; (**l**) Carvacrol; (**m**) Benzaldehyde; (**n**) Geraniol; (**o**) (1S)-(-)-verbenone; (**p**) Camphor; (**q**) (+)-pulegone.

Compared with the acetone control, house flies were significantly repelled by p-cymene at 0.1 μg/μL (*p* = 0.028), 10 μg/μL (*p* = 0.011), and 100 μg/μL (*p* = 0.028) (Figure 2a). For citronellic acid, the house flies were significantly repelled at high concentrations, 10 μg/μL (*p* = 0.011) and 100 μg/μL (*p* = 0.028), but not at low concentrations (Figure 2b). Estragole, (R)-(+)-Limonene, linalool, and eugenol were significantly repellent to the house flies only at the highest concentration, 100 μg/μL (*p* = 0.028) (Figure 2c–f). γ-Terpinene was significantly repellent only at the 10 μg/μL concentration (*p* = 0.028) (Figure 2g). Significant differences for thymol were observed at 10 μg/μL (*p* = 0.028) and 100 μg/μL (*p* = 0.028), which appeared to attract the house flies (Figure 2h). Additionally, the house flies were significantly attracted to (-)-carvone at 0.1 μg/μL (*p* = 0.011) (Figure 2i). There was no significant preference of house flies to (+)-fenchone (Figure 2j), methyl salicylate (Figure 2k), carvacrol (Figure 2l), benzaldehyde (Figure 2m), geraniol (Figure 2n), (1S)-(-)-verbenone (Figure 2o), camphor (Figure 2p), or (+)-pulegone (Figure 2q).

## 4. Discussion

One objective of our study was to evaluate the topical toxicity and repellency of 17 selected individual EOCs against adult house flies. Compared with some previous studies [34,35], our results showed that when only females were tested, the overall LD_50_ values were higher than those obtained when tests were performed with both males and females. Sukontason et al. [36] found that male house flies proved to be more susceptible than females to topical applications of eucalyptol. This is in accordance with several insecticide bioassay tests conducted on house flies. Mee et al. [37], Carriere [38], and Kaufman et al. [12] observed disproportionate survival between sexes: males were more susceptible to pesticides than females. Carriere [38] considered sexual size dimorphism to be the reason for differences in susceptibility by sex. In another study, house fly males weighed considerably less than females in every generation [12]. 

In female house flies, thymol, (+)-pulegone, eugenol, and carvacrol were the top four toxic components against female house flies at both 24- and 48 h posttreatment based on respective LD_50_ values. Thymol was the most toxic EOC among the 17 EOCs evaluated in this study using topical application. This result, although slightly higher, is similar to the LD_50_ values reported by Lee et al. [35] and Rice and Coats [34]. Some of the EOCs evaluated in this study had a higher LD_50_ value than the results in previous studies. This could be due to multiple factors such as fly strains, application methods, chemical resources, and experimental design. Besides the adult house flies, thymol was also reported to have high contact toxicity and fumigation toxicity to house fly larvae and pupa [22] as well as other pests such as mosquitoes [39]. Although thymol had the lowest slope, it is still a good candidate as an eco-friendly pesticide against house flies due to its low LD_50_ compared to the EOCs with a high slope, even if high mortality is required. 

No attempts were made to evaluate the resistance levels in the resistant strain of house flies from the USDA in Gainesville, FL. Resistance evaluations of house flies captured in the vicinity of Gainesville indicate the usual scenario, i.e., resistance continues to increase [27,40]. This is most likely attributed to the overuse of commonly available products. Although we were looking for a permethrin-resistant strain, what we had, according to publications, was a multi-resistant strain, most likely with resistance to permethrin, other pyrethroids and other non-pyrethroid pesticides [27]. The susceptible strain, also from the USDA in Gainesville, FL, is one of the few available susceptible strains. It has been in colony since the 1950s–1960s and has never been exposed to pesticides [27]. 

When comparing the results of the resistant and susceptible house fly strains, there were no significant differences except for the LD_50_ of (+)-pulegone and eugenol and LD_95_ of (+)-pulegone at 48 h posttreatment. These results indicated that no resistance had developed in the resistant house fly strain to the tested EOCs, at least for a short-term effect, while little to no resistance was observed for the longer-term effect. Therefore, these EOCs have the potential to provide an alternative management method for house flies that are resistant to permethrin and other pesticides. Cross-resistance has been indicated between certain EOs and permethrin in resistant house fly populations [41], but not with the EOCs used in our studies. 

The Pearson product–moment correlation was used to determine the correlation between toxicity and physical properties of the EOCs. For comparisons of boiling point, Log P, and density to toxicity, the R^2^ values and Pearson correlation coefficients were low, indicating poor relationships between toxicity and most physical properties. However, vapor pressure did correlate well with a log-corrected 24 h LD_50_ (Figure 1). This relationship is moderately strong (Pearson coefficient = 0.59) and statistically significant. As a positive Pearson correlation coefficient was observed, this correlation indicates that the most toxic terpenoids are also the least volatile (lowest vapor pressure). The increased toxicity due to lower-volatility constituents may demonstrate that select terpenoids penetrate the cuticle better if they do not volatilize well. Numerous groups have shown that essential oils act at various target sites within insects, exerting toxic effects (e.g., octopamine/tyramine receptors, acetylcholinesterase enzymes, GABA-gated chloride channels, etc.) [42,43,44,45]; therefore, lower volatility and more potential to penetrate the cuticle could be significant predictors of contact toxicity, as demonstrated in this study. 

We also investigated the relationship between the toxicological and chemical properties of EOCs using a Y-tube olfactometer. The treated and control filter paper strips were discarded and replaced after 10 flies (5 females and 5 males) were tested. The testing of 10 flies required 7–10 min, during which time less of the more volatile chemicals may have remained on the treated strip. We had no way to verify how much of the chemicals remained on the strip, and testing fewer flies to reduce the treatment time would have greatly prolonged the testing procedure.

Our olfactometer bioassay results indicated that p-cymene, citronellic acid, (R)-(+)-limonene, linalool, estragole, eugenol, and γ-terpinene were repellent, while thymol and (-)-carvone were attractive to house flies at certain concentrations. These EOCs, including the two displayed attractive properties, have been reported to exhibit repellent activities to other pests as well. P-cymene and (+)-limonene have been reported to exhibit repellent activity against a mosquito species, *Culex pipiens pallens* (L.) [46], and the rice weevil, *Sitophilus oryzae* (L.) [47], respectively. The German cockroach, *Blattella germanica* (L.) [48], and the yellow fever mosquito, *Ae. aegypti* [49], were also repelled by (+)-limonene. Linalool and eugenol, which were repellent to house flies at high concentrations in the current study, are also repellent to phlebotomine sand flies and mosquitoes [50] as well as the stored-product coleopterans *S. granarius* (L.), *S. zeamais* (Motschulsky), *Tribolium castaneum* (Herbst), and *Prostephanus truncatus* (Horn) [51].

Thymol has been reported to be a repellent to many insect species, such as *Anopheles stephensi* (Liston) [52], *Protophormia humanus capitis* (De Geer) [53], and *T. castaneum* [54]. The repellency of (-)-carvone to insects, such as *Ae. aegypti* [55] and *Protophormia terraenovae* (Robineau-Desvoidy) [56], has been reported previously. However, in our study, these two components were attractive to house flies (Figure 2h,i), a response that has not previously been reported. Similar attraction responses were observed in mosquitoes to EOCs by Hao et al. [57], where citronellal, linalool, citral, and geraniol were attractive at lower concentrations and repellent at higher concentrations to *Ae. albopictus* (Skuse). Naik et al. [58] also found a concentration-dependent behavioral response of the honeybee to nerol. Thus, the comparatively low concentrations tested in our study may be the reason why thymol and (-)-carvone showed attractiveness to house flies in our bioassay. However, the exact mechanisms of this phenomenon remain unclear.

This study illustrated that some individual EOCs are toxic and repellent to adult house flies. Earlier studies reported that many EOC blends have repellent activity to house flies. Our study is the first to demonstrate that seven of the seventeen individual EOCs also have significant repellency to house flies with attraction effects detected in two other EOCs. This screening of a wide variety of individual EOCs provides a stronger foundation of information for further research. By obtaining the LD_50_ and LD_95_ values and comparing these data with previous studies, we conclude that many plant EOCs are demonstrably insecticidal. Thymol and (-)-carvone, which were attractive to house flies, could be developed as natural baits to be used with traps. Given the high toxicity of thymol, it has the potential to be used as a lure and insecticide to attract and kill house flies. This will encourage further investigation into the topical toxicity and repellency of these compounds with susceptible strains and field-collected flies. Further field studies with these chemicals will provide further insights into the performance of biopesticides for house fly control and potential commercialization. 

## Figures and Tables

**Table 1:** LD_50_ and LD_95_ values at 24 h posttreatment produced when essential oil components were applied topically to resistant adult *Musca* domestica females.

Chemical	*n*	Slope ± SE	LD_50_ (95% CI) (µg/fly)	LD_95_ (95% CI) (µg/fly)	χ^2^
Thymol	500	1.8 ± 0.2	43.8 (34.1–55.6)	360.4 (221.0–823.7)	36.2
(+)-Pulegone	500	5.0 ± 0.6	73.0 (62.1–82.7)	155.6 (125.9–241.8)	46.7
Eugenol	500	5.3 ± 0.4	89.5 (71.5–108.1)	182.9 (144.7–285.5)	110.6
Carvacrol	500	3.4 ± 0.2	90.8 (77.2–106.5)	275.7 (216.6–390.8)	40.0
Citronellic acid	500	2.5 ± 0.2	93.4 (75.5–115.7)	430.1 (300.3–754.9)	44.6
Benzaldehyde	500	2.6 ± 0.2	94.7 (78.7–112.5)	392.4 (294.2–602.4)	29.4
Geraniol	500	2.7 ± 0.2	99.7 (85.7–116.4)	404.7 (311.0–582.9)	26.6
p-Cymene	600	3.1 ± 0.3	119.8 (106.1–133.4)	404.5 (342.7–502.7)	21.7
Estragole	500	3.3 ± 0.3	189.5 (161.4–222.5)	603.3 (460.4–922.1)	43.8
(-)-Carvone	500	4.8 ± 0.4	213.7 (184.6–242.0)	469.8 (396.8–605.8)	45.0
(R)-(+)-Limonene	500	2.1 ± 0.2	226.6 (185.1–286.6)	1208.5 (772.2–2751.4)	31.4
γ-Terpinene	500	4.4 ± 0.3	236.5 (216.2–256.8)	556.9 (486.9–667.8)	21.8
Linalool	600	2.2 ± 0.2	238.1 (201.7–282.2)	1322.1 (943.4–2169.8)	37.6
Methyl salicylate	500	5.0 ± 0.4	260.7 (237.4–285.5)	557.9 (479.6–694.1)	27.9
(+)-Fenchone	700	3.8 ± 0.3	405.1 (359.3–457.7)	1094.9 (869.0–1590.4)	75.5
(1S)-(-)-Verbenone	600	5.0 ± 0.4	426.7 (399.9–454.8)	909.3 (802.0–1081.1)	28.1
Camphor	500	9.2 ± 0. 7	512.1 (486.6–539.3)	774.9 (709.4–883.0)	33.6

**Table 2:** LD_50_ and LD_95_ values at 48 h posttreatment produced when essential oil components were applied topically to resistant adult *Musca domestica* females.

Chemical	*n*	Slope ± SE	LD_50_ (95% CI) (µg/fly)	LD_95_ (95% CI) (µg/fly)	χ^2^
Thymol	500	1.9 ± 0.2	41.1 (32.1–51.8)	317.2 (199.8–686.4)	36.5
(+)-Pulegone	500	8.8 ± 0.9	68.2 (63.0–73.0)	104.8 (94.7–123.4)	69.0
Eugenol	500	5.7 ± 0.4	78.5 (64.3–93.5)	153.1 (123.6–222.6)	93.7
Carvacrol	500	3.5 ± 0.2	80.6 (68.3–95. 0)	237.7 (186.1–340.2)	42.7
Geraniol	500	2.6 ± 0.2	85.6 (71.2–102.7)	373.0 (274.2–586.6)	34.6
Citronellic acid	500	2.4 ± 0.2	85.8 (69.9–105.0)	430.3 (302.6–736.6)	37.9
Benzaldehyde	500	2.6 ± 0.2	86.8 (71.8–103.7)	381.2 (283.1–594.2)	29.9
p-Cymene	600	3.1 ± 0.3	111.5 (98.5–124.4)	375.9 (319.0–465.9)	21.5
Estragole	500	2.9 ± 0.2	164.8 (135.6–199.1)	614.8 (445.6–1047.3)	51.1
(-)-Carvone	500	4.8 ± 0.4	190.4 (166.4–213.9)	422.0 (360.9–527.6)	35.2
Linalool	600	2.4 ± 0.2	209.7 (179.7–244.5)	1042.4 (778.1–1580.2)	35.3
(R)-(+)-Limonene	500	1.9 ± 0.2	213.4 (162.0–275.2)	1356.9 (819.0–3558.8)	32.4
γ-Terpinene	500	4.4 ± 0.3	221.6 (200.7–242.)	526.5 (457.1–628.6)	24.8
Methyl salicylate	500	4.5 ± 0.4	238.7 (221.4–256.5)	549.5 (485.4–646.0)	17.0
(+)-Fenchone	700	3.5 ± 0.3	385.3 (341.3–434.9)	1138.6 (898.4–1653.3)	66.6
(1S)-(-)-Verbenone	600	5.2 ± 0.4	410.0 (383.3–437.3)	853.9 (756.2–1009.7)	30.5
Camphor	500	8.8 ± 0.7	478.0 (454.3–502.3)	734.7 (675.8–828.0)	30.7

**Table 3:** LD_50_ and LD_95_ values at 24 h posttreatment produced when essential oil components were applied topically to susceptible adult *Musca domestic* females.

Chemical	*n*	Slope ± SE	LD_50_ (95% CI) (µg/fly)	LD_95_ (95% CI) (µg/fly)	χ^2^
Thymol	150	2.0 ± 0.3	35.2 (19.4–59.7)	227.5 (110.6–1659.9)	5.7
(+)-Pulegone	125	2.5 ± 0.5	56.5 (43.3–75.3)	262.9 (156.0–912.9)	2.7
Eugenol	125	2.5 ± 0.5	60.9 (45.7–79.9)	276.2 (174.9–703.2)	2.5
Carvacrol	150	2.1 ± 0.4	77.4 (46.8–122.4)	463.0 (232.4–3641.1)	4.5
Citronellic acid	150	2.2 ± 0.4	92.0 (45.8–165.4)	530.1 (249.8–7648.1)	6.3
Geraniol	150	2.4 ± 0.5	93.1 (70.5–121.9)	463.8 (284.2–1312.2)	2.9

**Table 4:** LD_50_ and LD_95_ values at 48 h posttreatment produced when essential oil components were applied topically to susceptible adult *Musca domestica* females.

Chemical	*n*	Slope ± SE	LD_50_ (95% CI) (µg/fly)	LD_95_ (95% CI) (µg/fly)	χ^2^
Thymol	150	1.8 ± 0.3	29.2 (13.8–53.2)	244.1 (106.0–3264.8)	6.2
(+)-Pulegone	125	2.3 ± 0.4	38.8 (28.4–50.5)	207.2 (127.9–572.3)	1.6
Eugenol	125	2.3 ± 0.4	45.1 (30.5–63.3)	232.4 (120.4–1922.3)	3.1
Carvacrol	150	2.0 ± 0.4	65.7 (48.1–87.2)	430.0 (258.5–1132.0)	2.7
Citronellic acid	150	2.4 ± 0.3	75.5 (55.3–100.7)	327.1 (194.1–4273.7)	3.7
Geraniol	150	2.6 ± 0.5	75.7 (48.5–107.6)	479.4 (292.7–1175.5)	6.9

## Data Availability

The data reported in this study are available upon request.

## Supplementary Materials

The following supporting information can be downloaded at: https://www.mdpi.com/article/10.3390/insects15060384/s1, Table S1. Contact toxicity to insects of the 17 essential oil components used in our studies; Table S2. Chemical structure and properties of essential oils and individual components. References [59,60,61,62,63,64,65,66,67,68,69] are in Supplementary Materials.
