# Supplementary material for: Topical Toxicity and Repellency Profiles of 17 Essential Oil Components against Insecticide-Resistant and Susceptible Strains of Adult Musca domestica (Diptera: Muscidae)
Source: Insects. 2024 May 24;15(6):384. doi: 10.3390/insects15060384 (PMC11203968; doi:10.3390/insects15060384)
Supplement: Supplementary file 1 [file insects-15-00384-s001.zip › insects-3003346-supplementary.pdf]

Table S1. Contact toxicity to insects of the 17 essential oil components used in our studies.

Essential oil	Insect	Scientific Name	LD_{50}/LC_{50}	Reference
R-Limonene	House fly	*Musca domestica* (Diptera: Muscidae)	68 µg/fly	[35]
	Maize weevil	*Sitophilus zeamais* (Coleoptera: Curculionidae)	29.86 µg/ insect	[59]
	Red flour beetle	*Tribolium castaneum* (Coleoptera: Tenebrionidae)	20.14 µg/insect	[59]
	Rice weevil	*Sitophilus oryzae* (Coleoptera: Curculionidae)	477.19 µg/cm^2	[60]
	Red flour beetle	*Tribolium castaneum*	478.46 µg/cm^2	[60]
	Tobacco cutworm	*Spodoptera litura* (Leidoptera: Noctuidae)	273.7 µg/insect	[61]
Carvacrol	House fly	*Musca domestica*	92 µg/fly	[35]
	House fly	*Musca domestica*	63 µg/fly	[34]
	German cockroach	*Blattella germanica* (Blattodea: Ectobiidae)	0.186 mg/insect	[62]
	Tobacco cutworm	*Spodoptera litura*	42.7 µg/insect	[61]
p-Cymene	House fly	*Musca domestica*	282.1/insect	[35]
Geraniol	House fly	*Musca domestica*	73 µg/fly	[35]
	House fly	*Musca domestica*	103 µg/fly	[34]
	House fly	*Musca domestica*	45.63µg/fly	[63]
	German cockroach	*Blattella germanica*	0.832 mg/insect	[62]
	Red flour beetle	*Tribolium castaneum*	179.35 µg/cm^2	[60]
	Rive weevil	*Sitophilus oryzae*	28.76 µg/cm^2	[60]
Linalool	House fly	*Musca domestica*	116 µg/fly	[35]
	House fly	*Musca domestica*	0.04 µg/fly	
	House fly	*Musca domestica*	106.88µg/fly	[63]
	House fly	*Musca domestica*	189 µg/fly	[34]
	Rice weevil	*Sitophilus oryzae*	66.74 µg/cm^2	[60]
	Red flour beetle	*Tribolium castaneum*	105.63 µg/cm^2	[60]
	Maize weevil	*Sitophilus zeamais*	10.46 µg/insect	[64]

Essential oil	Insect	Scientific Name	LD_{50}/LC_{50}	Reference
Eugenol	Maize weevil	*Sitophilus zeamais*	34 µg/insect	[65]
	Red flour beetle	*Tribolium castaneum*	174 µg/insect	[65]
	House fly	*Musca domestica*	77 µg/fly	[35]
	Tobacco cutworm	*Spodoptera litura*	157.6 µg/insect	[61]
	Wireworm	*Agriotes obscurus* (Coleoptera: Elateridae)	516 µg/insect	[66]
	German cockroach	*Blattella germanica*	0.294 mg/insect	[62]
Thymol	House fly	*Musca domestica*	29 µg/fly	[35]
	House fly	*Musca domestica*	33 µg/fly	[34]
	Tobacco cutworm	*Spodoptera litura*	25.4 µg/insect	[61]
	Wireworm	*Agriotes obscurus*	195.5 µg/insect	[66]
	German cockroach	*Blattella germanica*	0.122 mg/insect	[62]
(+)-Pulegone	House fly	*Musca domestica*	39 µg/fly	[35]
	House fly	*Musca domestica*	78 µg/fly	[34]
	Tobacco cutworm	*Spodoptera litura*	51.6 µg/insect	[61]
Citronellic acid	House fly	*Musca domestica*	32 µg/fly	[35]
	House fly	*Musca domestica*	43 µg/fly	[34]
	German cockroach	*Blattella germanica*	0.491 mg/insect	[62]
l-Fenchone	House fly	*Musca domestica*	222 µg/fly	[35]
	Rice weevil	*Sitophilus oryzae*	291.80 $µg/cm^2$	[60]
	Red flour beetle	*Tribolium castaneum*	179.49 $µg/cm^2$	[60]
γ-Terpinene	House fly	*Musca domestica*	214 µg/fly	[35]
(-)-Carvone	House fly	*Musca domestica*	102 µg/fly	[35]
	House fly	*Musca domestica*	173 µg/fly	[34]
	Rice weevil	*Sitophilus oryzae*	28.17 $µg/cm^2$	[60]
	Red flour beetle	*Tribolium castaneum*	19.80 $µg/cm^2$	[60]
(-)-Verbenone	House fly	*Musca domestica*	176 µg/fly	[34]

Essential oil	Insect	Scientific Name	LD_{50}/LC_{50}	Reference
	House fly	*Musca domestica*	247 µg/fly	[35]
Estragole	Maize weevil	*Sitophilus zeamais*	17.63 µg/insect	[64]
	Maize weevil	*Sitophilus zeamais*	39 µg/insect	[65]
	Red flour beetle	*Tribolium castaneum*	73 µg/insect	[65]
Camphor	Cigarette beetle	*Lasioderma serricorne* (Coleoptera: Ptinidae)	11.30 µg/insect	[67]
	Red flour beetle	*Tribolium castaneum*	54.21 µg/insect	[67]
	Rice weevil	*Sitophilus oryzae*	>500 µg/cm^2	[60]
	Red flour beetle	*Tribolium castaneum*	>500 µg/cm^2	[60]
	Maize weevil	*Sitophilus zeamais*	137 µg/mg	[19]
	Red flour beetle	*Tribolium castaneum*	887 µg/mg	[19]
Methyl salicylate	Yellow fever mosquito	*Aedes aegypti* (Diptera: Culicidae)	39700 µg/g	[68]
	African malaria mosquito	*Anopheles gambiae* (Diptera: Culicidae)	11100 µg/g	[68]
Benzaldehyde	Asian tiger mosquito	*Aedes albopictus* (Diptera: Culicidae)	47.0 µg/ml	[69]

Table S2. Chemical structure and properties of essential oils and individual components.

Chemical	Density (g/mL at 25°C)	Assay %	Boling point (°C)	Log P	Log (vapor pressure) Log10 (mmHg)
**Alcohols**					
Geraniol	0.879	98.0	229	2.89	-1.52
Linalool	0.870	97.0	194	2.68	-0.80
**Phenols**					
Carvacrol	0.976	98.0	236	3.20	-1.90
Eugenol	1.067	99.0	254	2.66	-1.65
Thymol	0.965	99.0	232	3.16	-2.66
**Ketone**					
(-)-Carvone	0.960	99.0	228	2.77	-0.99
(+)-Fenchone	0.945	99.5	63	2.54	-0.09
(+)-Pulegone	0.935	99.5	223	2.36	-0.91
(1S)-(-)-verbenone	0.975	93.0	227	2.30	-0.60
Camphor	0.992	96.0	204	2.85	-0.19
**Aldehyde**					
Benzaldehyde	1.045	99.5	178	1.60	0.10
**Acid**					
Citronellic acid	0.923	98.0	121	2.88	-2.74
**Hydrocarbons**					
p-Cymene	0.860	99.0	176	4.17	0.16
(R)-(+)-limonene	0.842	99.5	176	4.50	0.19
γ-Terpinene	0.850	99.5	182	4.36	0.04
**Ether**					
Estragole	0.965	99.5	215	3.23	-0.93
**Ester**					
Methyl salicylate	1.174	99.0	222	2.07	-1.47
